# Buyang huanwu decoction promotes angiogenesis via vascular endothelial growth factor receptor-2 activation through the PI3K/Akt pathway in a mouse model of intracerebral hemorrhage

**DOI:** 10.1186/s12906-015-0605-8

**Published:** 2015-03-28

**Authors:** Han-Jin Cui, A-Li Yang, Hua-Jun Zhou, Cong Wang, Jie-Kun Luo, Yuan Lin, Yan-Xia Zong, Tao Tang

**Affiliations:** Institute of integrative medicine, Xiangya Hospital, Central South University, Changsha, 410008 Hunan China; Institute of neurology, Xiangya Hospital, Central South University, Changsha, 410008 Hunan China; Key Lab of Chinese Gan of SATCM, Changsha, 410008 Hunan China; Institute of Neurology, The First College of Clinical Medical Sciences, China Three Gorges University, Yichang, 443003 Hubei China

**Keywords:** Intracerebral hemorrhage, Angiogenesis, Vascular endothelial growth factor receptor-2, Phosphorylation, PI3K/Akt, Buyang huanwu decoction

## Abstract

**Background:**

Intracerebral hemorrhage (ICH) is a fatal subtype of stroke that lacks effective treatments. Angiogenesis following ICH is an important response mediating brain recovery and repair. Phosphorylation of vascular endothelial growth factor receptor 2 (pVEGFR2) via PI3K/Akt signaling plays a key role in mediating cellular processes involved in repair, such as mitogenesis, angiogenesis, and vascular permeability. This study aimed to investigate the potential effects of Buyang Huanwu Decoction (BYHWD), a traditional Chinese medicine formula, on angiogenesis by VEGFR2 activation through the phosphatidylinositol 3 kinase (PI3K)/Akt signaling pathway in a mouse model of ICH.

**Methods:**

Adult male Kunming mice (n = 50) were randomly assigned into sham and ICH-operated groups and treated with one of the followings SU5416 (VEGFR2 inhibitor), BYHWT and BYHWT + SU5416. ICH was induced in mice by injecting collagenase (type VII) into the right globus pallidus of the mouse brain. BYHWD (4.36 g/kg) was administrated in mice by intragastric infusion. Neurological function was evaluated in mice by a modified Neurological Severity Scores (mNSS) as well as corner turn and foot-fault tests. Angiogenesis was examined by intraperitoneal injection of 5-bromodeoxyuridine (BrdU) in mice to quantify new brain vessel growth. SU5416 treatment and assessment of VEGFR2 phosphorylation as well as alterations in PI3K/Akt signaling were performed to determine whether the effect of BYHWD on angiogenesis was partly mediated by phosphorylation of VEGFR2 via the PI3K/Akt signaling pathway.

**Results:**

We show that BYHWD treated mice exhibited (i) significantly better recovery from neurological dysfunction, (ii) increased BrdU^+^ nuclei in vWF^+^ dilated brain vessels and (iii) higher VEGFR2 phosphorylation immunoreactivity in brain microvessels (*P* <0.05), (iv) higher expression of PI3K and pAkt at the protein level (P <0.05) when compared to untreated ICH mice. These beneficial effects were reversed by SU5416 (P <0.05).

**Conclusions:**

BYHWD promoted neurological recovery and angiogenesis after ICH in mice by enhancing VEGFR2 phosphorylation through the PI3K/Akt signaling pathway.

## Background

Spontaneous intracerebral hemorrhage (ICH) is one of the most lethal types of stroke [1] with the highest mortality and morbidity [2]. Conventional therapies for ICH consist of hematoma removal, edema attenuation and intracranial pressure reduction. However, the effectiveness of ICH therapy is far from satisfactory [3]. It is therefore important and necessary to explore new strategies for ICH treatment.

Recent studies on pathophysiological mechanisms of ICH impairment mainly focus on secondary brain injury and neuronal death caused by inflammation iron accumulation. Inflammation is mediated by inflammatory cells (blood-derived leukocytes, macrophages, microglia, astrocytes, mast cells) activation and their local release of cytokines, proteases, prostaglandins, proteases and so on [4,5]. Excessive iron accumulation after ICH due to heme degradation can stimulate reactive oxygen species (ROS) formation causing neurotoxicity and neurodegeneration [6]. Despite these impairment factors, there are also endogenous mechanisms of brain self-repair after stroke, which include angiogenesis.

Normal brain function requires unrestricted blood flow to supply glucose and oxygen to neurons. Following a damaging ICH event, new microvasculature formation is required for brain recovery by promoting oxygen and metabolite exchange and removing necrotic debris [7,8]. Angiogenesis refers to the establishment of new vessels from pre-existing vasculature by such processes as sprouting, pruning and intussusception. It starts with stimulation of endothelial cells (ECs) that line the luminal surface of blood vessels, and occurs under both physiological and pathological conditions [9,10]. During angiogenesis, a highly regulated process, the most potent pro-angiogenic signaling cascades are initiated by vascular endothelial growth factor (VEGF) binding to EC-resident VEGF receptors (VEGFR) like VEGFR1, VEGFR2 and VEGFR3 [9]. Of these, VEGFR2 transduces angiogenic signals after binding VEGF, while VEGFR1 transduces only weak signals for ECs growth and survival, and VEGFR3 is generally restricted to lymphatic endothelia [11]. VEGF expression has been evaluated and shown to be an important component of the response after brain injury [12]. We have previously demonstrated that angiogenesis can occur following ICH in brains, and are able to specifically detect VEGF and VEGFR2 around the hematoma [13].

VEGF binding to VEGFR2 leads to the receptor dimerization, protein kinase activation, autophosphorylation, and finally, the initiation of several signaling cascades [14]. Amongst the endothelial cell signaling pathways, PI3K/Akt is known to be a major regulator of proliferation, migration and survival of ECs as well as vascular permeability. VEGFR2 activation is believed to be the “switch” that activates the downstream PI3K/Akt signaling cascade [15]. SU5416, a potent selective inhibitor of VEGFR2 tyrosine kinase catalysis, 3-[2, 4- dimethylpyrrol-5-yl methylidenyl] -2-indolinone, has been shown to reduce tumor angiogenesis and growth in animal models [16-18].

Traditional Chinese medicine (TCM) has been shown to have positive effects in stroke [19]. As a classical TCM prescription for stroke, Buyang Huanwu Decoction (BYHWD) shows substantial neuroprotective, pro-angiogenic and function-improving effects in animal models of focal cerebral ischemia [20-22], while also possessing desirable clinical efficacy for both hemorrhagic and ischemic stroke [23]. Furthermore, we have shown previously that BYHWD could upregulate VEGF and VEGFR2 after ICH [24]. Since VEGFR2 activation can efficiently stimulate angiogenesis [25], we hypothesized that BYHWD could likewise promote angiogenesis in the ICH model through VEGFR2 stimulation. Therefore, we were interested in studying the effects of BYHWD on the recovery of the brain after hemorrhagic injury as well as VEGFR2 phosphorylation and its signaling pathway mechanisms.

## Methods

### Animal preparation

Studies were carried out on adult male Kunming mice (25–30 g, 8–10 weeks of age) obtained from the Experimental Animal Science Center of Central South University (CSU). All animals were housed under identical conditions (room temperature at 25°C, 12 h light–dark cycle) and allowed free access to food and water. The experimental protocol was performed in compliance with guidelines of CSU and the NIH Guide for the Care and Use of Laboratory Animals (NIH Publications No. 80–23) and was approved by the Institutional Animal Care and Use Committee of CSU (2010-xy-0106).

### BYHWD preparation

According to the original prescription from the “TCM Prescriptions Dictionary”, BYHWD consists of seven Chinese herbs (Table 1). All dried crude herbs were purchased from the Chinese Medicinal Pharmacy of Xiangya Hospital, Changsha, China. Voucher specimens were prepared and deposited at the Laboratory of Institute of Integrative Medicine, Central South University. To maintain the consistency of the herbal chemical ingredients, all components were obtained from the original sources and extracted according to standards listed in the National Pharmacopoeia of China. The herbs were cut, mixed, and decocted by boiling in distilled water at 100°C for 30 min. The solution was then freeze-dried under vacuum, and ground into powder (yield: 14.3%). The powder was dissolved in distilled water to a final concentration of 0.132 g/ml.

**Table 1:** **Components of the buyang huanwu decoction**

**Latin name**	**Family**	**English name**	**Chinese name**	**Part used**	**Batch number**
Radix astragali	Leguminosae	Astragalusmongholicus	Huang qi	Root	091201
Radix paeoniaeRubra	Ranunculaceae	Red peony root	Chi shao	Root	091214
Radix angelicaeSinensis	Umbelliferae	Angelica root	Dang gui	Root	091206
RhizomaLigusticiChuanxiong	Umbelliferae	Sichuan lovage rhizome	Chuanxiong	Root	100304
FlosCarthami	Feverfew	Carthamustinctorius	Hong hua	Flower	100329
Semen persicae	Rosaceae	Peach seed	Tao ren	Seed	091218
PheretimaAspergillum	Megascolecidae	Earthworm (Lumbricus)	Di long	Whole animal	091222

In the order listed above, the herbs were combined in a 60:6:4.5:3:3:3 ratio (dry weight).

### ICH induction

After fasting overnight, the mice were anesthetized with 5% chloral hydrate (300 mg/kg IP) and then fixed on a stereotaxic frame (Stoelting Co., Chicago, IL, USA) in the prone position. Following a scalp incision and drilling of a small cranial bur hole, 0.075 U of collagenase (type VII) in 0.5 μl 0.9% sterile saline was slowly injected into the right globus pallidus (0.5 mm posterior, 2.0 mm lateral to bregma and 4.0 mm ventral to cortical surface) of the mouse brain, using a 1-μl Hamilton syringe. The infusion was administered over 2 min, with the needle kept in place for an additional 5 minutes thereafter. In the sham-operated group, 0.5 μl 0.9% sterile saline without collagenase was injected into the same site.

### Experimental protocol

Mice were randomly assigned to both sham and ICH operated groups and treated with one of the followings: SU5416, BYHWT and BYHWT + SU5416 (n = 10, per group). Twenty-four hours after ICH induction, 5-bromodeoxyuridine (BrdU, Roche, Indianapolis, IN, USA) was given (50 mg/kg/d) by intraperitoneal injection. The SU5416-treated and BYHWT + SU5416-treated groups were administrated with SU5416 (10 mg/kg, Santa Cruz, Dallas, Texas, USA) in distilled water (DW) containing 1% dimethyl sulfoxide (DMSO, Calbiochem, La Jolla, CA, USA) by intraperitoneal injection. Control groups received 1% DMSO in their respective cocktails. BYHWD (4.36 g/kg) was given to BYHWD-treated and BYHWT + SU5416-treated groups intragastrically. Equal volume of DW was given to all other groups. The dose of BYHWD of 4.36 g/kg was chosen from the preliminary tests for the recovery of motor function in ICH animals (data not shown). Administration of drug treatments was performed 24 h after ICH induction, once a day for 7 days.

### Behavioral tests

Behavioral tests were carried out at day 1, 3, 7 after ICH. Two observers, blinded to the experimental design, scored the animals independently and the scores were averaged. The tests were composed of a modified Neurological Severity Score (mNSS) [26], corner turn test [27] and foot-fault test [28].

1. Neurological score: An eighteen point neurological score was employed: (normal score, 0; maximal deficit score, 18). For severity scores of injury, 1 score point is awarded for the inability to perform the test or for the lack of a tested reflex; thus, the higher score, the more severe the injury [26].
2. Corner turn test: Mice were allowed to proceed into a corner, the angle of which was 30 degrees. To exit the corner, individual mice could turn either left or right, and the direction taken was then recorded. This was repeated 10 to 15 times per animal, with at least 30 seconds between trials, and the percentage of right turns was calculated [27].
3. Foot-fault test: Mice were tested for placement dysfunction of forelimbs with the modified foot-fault test. Animals were set on an elevated grid surface (85.8 × 2.5 cm^2^, with grids of different sizes) and placed their paws on the wire while moving along the grid. With each weight-bearing step, the paw may fall or slip between the wire. Each such event was recorded as a foot fault. The total number of steps (movement of each forelimb) that the mice used to cross the grid was counted, and the total numbers of foot faults for the left forelimb were recorded. Data are presented as the percentage of foot faults per the total number of steps [28].

### Specimen preparation

Randomly chosen animals from each group were deeply anesthetized with chloral hydrate (300 mg/kg). For immunohistochemistry, animals were transcardially perfused with 0.9% saline followed by 250 ml ice-cold 4% paraformaldehyde in 0.1 M phosphate buffer (PB, pH 7.4). The excised brains were post-fixed in the same fixative for 2 h, then transferred to 20% and then 30% sucrose in 0.1 M PB (pH 7.4) sequentially at 4°C until sinking. Brains were cut into 30 μm coronal sections at −20°C with a cryostat (CM1900, Leica, Germany), some of which were collected in 0.01 M phosphate-buffered saline (pH 7.4) and stored at 4°C.

For Western blot analysis, mice were transcardially perfused with ice-cold 0.9% saline and then sacrificed by decapitation. The brains were immediately removed, and the tissues in the striatum, adjacent to the hematoma without the needle track, were dissected and stored in liquid nitrogen tank.

### Immunohistochemical analysis

To detect proliferating cerebral microvascular ECs, immunohistochemical double-staining was performed. Sections were pretreated to denature DNA as follows: after immersion in 50% formamide/2× saline-sodium citrate buffer (SSC, pH 7) at 65°C for 2 h, sections were washed in 2× SSC for 10 min then incubated in 2 N HCl at 37°C for 30 min. After nonspecific antigen blocking in 5% bovine serum albumin (BSA, Sigma-Aldrich, St. Louis, MO, USA), sections were incubated with a mouse anti-BrdU (1:200, Neomarker, Fremont, CA, USA) at 4°C overnight, then with biotinylated anti-mouse IgG (1:200, Vector Laboratories, Burlingame, CA, USA) for 1 h at 37°C, followed by avidin-biotin-peroxidase complex (ABC) (1:100, Vector) for 1 h at 37°C. Immunoreactivity was visualized with diaminobenzidine (DAB, Boster Biotech, Wuhan China). For labeling cerebral EC’s, we used a rabbit anti-von Willebrand factor (vWF, 1:400, Chemicon International, Temecula, CA, USA) primary antibody in concert with ammonium nickel sulfate to enhance visualization. BrdU^+^/vWF^+^ nuclei close to the hematoma were counted in four 250 × 250 μm areas in five sections through the stroke for each animal at × 40 objective magnification using image J analysis software, by a researcher blind to the experimental design as previously described. Data were presented as the number of nuclei per mm^2^ (N/mm^2^).

For expression of phospho (p)-VEGFR2, sections were brought to room temperature and incubated in 3% H_2_O_2_ for 15 min. After washing sections 3 times in phosphate-buffered saline for 5 min each, non-specific binding was blocked in 5% bovine serum albumin (Sigma Aldrich) for 1 h at 37°C. Sections were then incubated overnight at 4°C with a pVEGFR2 rabbit mAb (diluted 1:100, Cell Signaling Technology, Boston, MA, USA), with a biotinylated anti–rabbit immunoglobulin G (1:200) for 2 h, and then with avidin-biotin-peroxidase complex (1:100, Vector) for 1 h at 37°C. Immunoreactivity was visualized with diaminobenzidine (DAB, Boster Biotech). Areas of immunoreactivity were measured using Qwin software to determine gray scale.

To determine whether pVEGFR2 was expressed in ECs, immunofluorescence double labeling was performed. Tissue sections were first incubated at 4°C for 48 h with a mixture of 2 primary antibodies: mouse anti–vWF (1:200, Chemicon Int) and either of the rabbit anti-phospho-VEGFR2 (1:100). The following secondary antibodies were then used: fluorescein isothiocyanate–conjugated goat anti–mouse antibody (1:50, Santa Cruz Biotech, Santa Cruz, CA, USA) for vWF and rhodamine-conjugated donkey anti-rabbit antibody (1:50, Santa Cruz) for pVEGFR2 detection. For a negative control, 1% bovine serum albumin was used instead of the primary antibody. These sections were scanned using a laser confocal microscope (LSM-510, Zeiss).

### Western blot assay

Tissues from the right globus pallidus were homogenized in RIPA buffer containing 50 mM Tris–HCl (pH 7.4), 150 mM NaCl, 1% sodium deoxycholate, 1% NP-40, 0.1% lauryl sodium sulfate, 1 mM phenylmethylsulfonyl fluoride (PMSF), and 1% inhibitor cocktail (BioBasic Inc, Markham, ON, Canada), and the homogenate was centrifuged at 14,000 *g* for 30 min. Proteins (50 μg) from the supernatant of each sample were then separated by SDS-PAGE and transferred onto polyvinylidene difluoride membranes, which were subsequently blocked in TBST buffer containing 20 mMTris–HCl, 5% skim milk, 150 mM NaCl, and 0.05% Tween 20 (pH 7.5) for 2 h at room temperature. Membranes were incubated with either goat anti-p-VEGFR2 (1:200, Santa Cruz Biotech), rabbit-anti-VEGFR2 (1:1000, Abcam, Cambridge, UK), rabbit anti-PI3K (1:2000, Millipore-Merck, Darmstadt, GER), rabbit anti-Akt (1:2000, Cell Signaling Tech), rabbit anti-p-Akt (1:2000, Cell Signaling Tech) or mouse anti-actin (1:5000, Abcam) antibodies for overnight at 4°C. Subsequently, membranes were incubated with horseradish peroxidase-conjugated anti-goat IgG (1:5000, Promega, Madison, WI, USA), anti-rabbit IgG antibody (1:5000, Promega) or anti-mouse IgG (1:5000, Promega) antibodies for 2 h at room temperature. The immunopositive bands were visualized by chemiluminescence detection using SuperSignalWest Pico Chemilumescent Substrate (Thermo) and a Bio-Rad ChemiDoc XRS digital documentation system (Bio-Rad, Hercules, CA, USA). The optical density (OD) of each band was normalized against that of β-actin.

### Statistical analysis

All data in this study are presented as mean ± standard deviation (SD). Data were analyzed with Student’s t test and one-way analysis of variance (ANOVA), followed by Scheffe’s post hoc test. Differences were considered significant at *P* <0.05.

## Results

### Effect of BYHWD on the neurological outcomes of ICH mice

**Figure 1:**
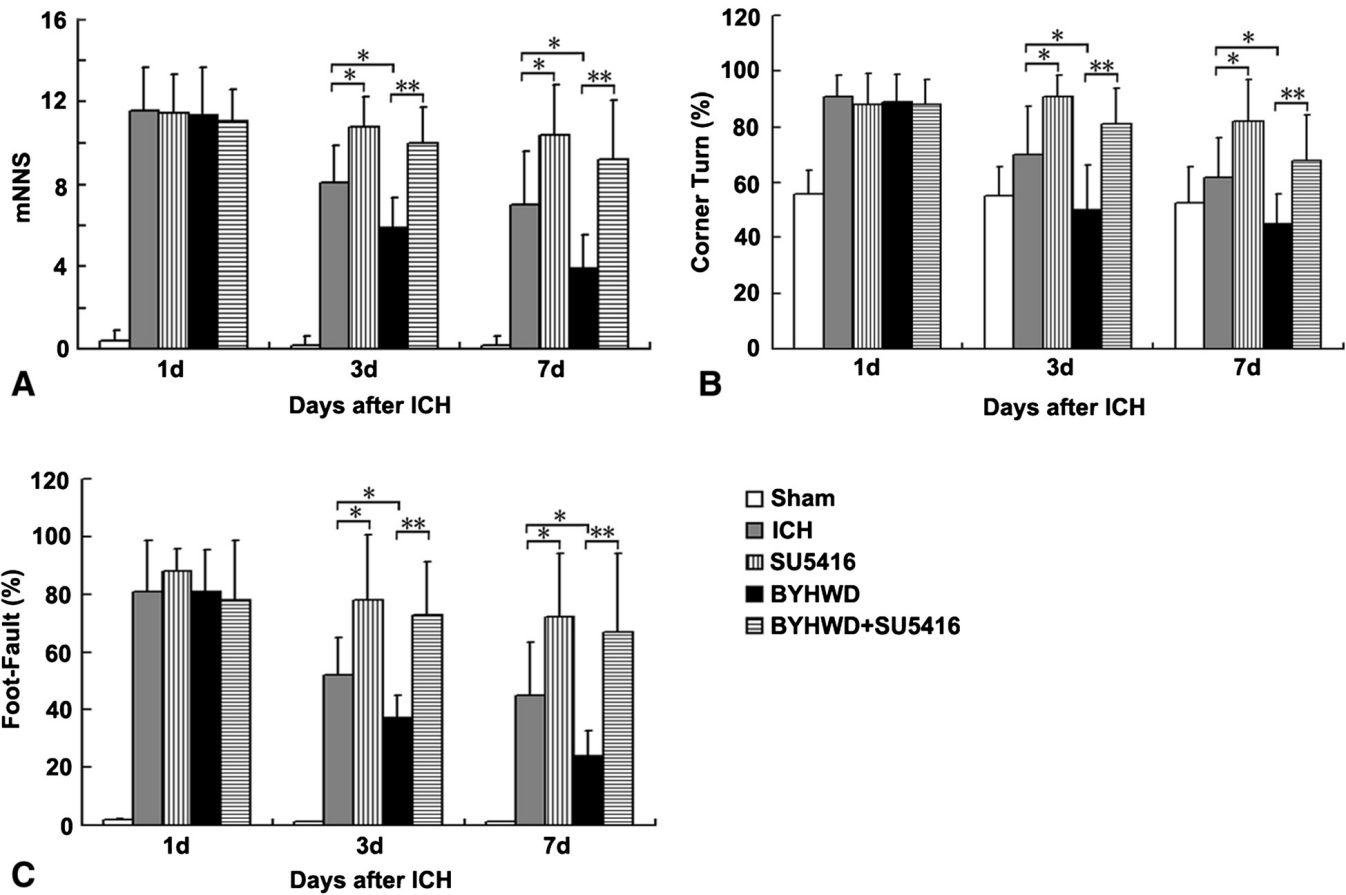
**Effect of BYHWD on neurological outcomes in mice after ICH.** Mice in the ICH group and BYHWD-treated group (3 d and 7 d) showed a significantly lower mNSS **(A)** as well as better performance in corner turn **(B)** and foot-fault **(C)** tests than their inhibitor-treated counterparts. Similar differences were found between BYHWD-treated group and ICH group. The percentage of right turns **(B)** and foot faults **(C)** from each group was compared with the total number of steps. Data represent the mean ± SD, n = 10. *, *P* <0.05; **, *P* <0.01). Data were analyzed with Student’s t test and one-way analysis of variance (ANOVA), followed by Scheffe’s post hoc test.

On the first day, all four groups subjected to collagenase-induced ICH showed similar neurological deficits, while the sham-operated animals were almost free of neurological impairments. Evaluation of mNSS at later time points revealed that the neurological status of both the ICH group and BYHWD-treated group improved over time (a decrease in the mNSS) with the former significantly higher than the latter (more than 2 points higher at day 3 and 7, *P* <0.05). However, the scores were significantly (*P* <0.05) higher in the SU5416-treated and BYHWD + SU5416-treated group compared with those in the ICH group (about 3 points higher at day 3 and 7) and BYHWD-treated group (more than 4 points higher at day 3 and 7), respectively (Figure 1A, *P* <0.05, *P* <0.01).

At day 3, the ICH group (about 20%) and BYHWD-treated group (about 30%) performed significantly better in the corner turn test than their inhibitor-treated counterparts, respectively (*P* <0.05, *P* <0.01). BYHWD-treated mice significantly outperformed ICH mice (about 20% lower at day 3 and 7, respectively) (Figure 1B, *P* <0.05). Similarly, BYHWD-treated mice significantly outperformed ICH mice in the foot-fault test (about 20% lower at day 3 and 7, respectively). Treatment with SU5416 worsened the response of the foot-fault test in BYHWD-treated (about 40% higher at day 3 and 7, respectively) and ICH mice (about 30% higher at day 3 and 7, respectively) (Figure 1C, *P* <0.01, *P* <0.05).

### BYHWD promoted angiogenesis via upregulating pVEGFR2

**Figure 2:**
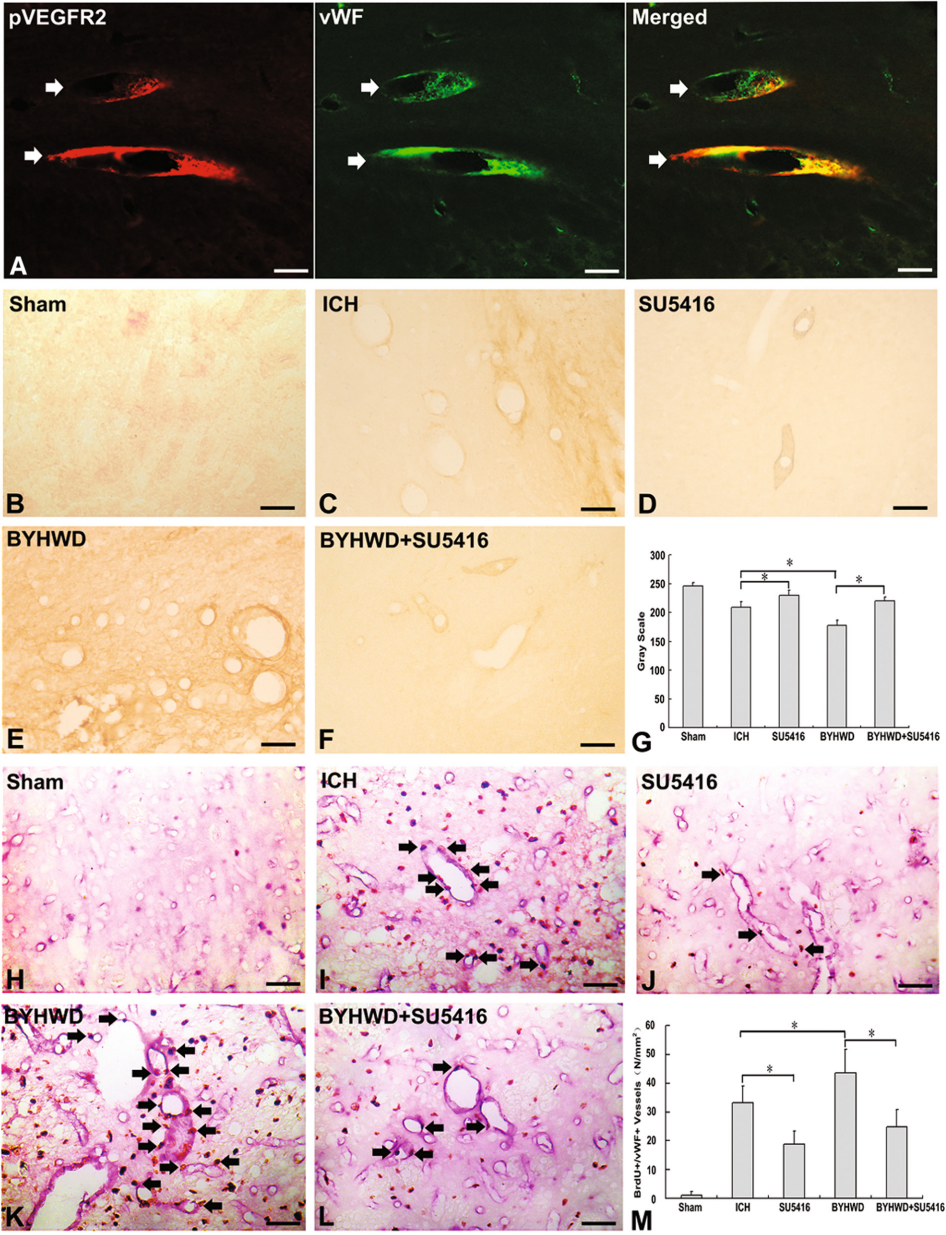
**BYHWD upregulates pVEGFR2 expression and angiogenesis following ICH.** Double-labeling revealed that immunoreactivity for pVEGFR2 (red, arrow) overlapped with vWF^+^ cells (green, arrow) **(A)**. pVEGFR2-positive microvessels and BrdU^+^ nuclei (brown, arrow) were shown in vWF^+^ vessels (blue) in either hemisphere of the sham-operated animals **(B and H)**, ICH group **(C and I)**, SU5416-treated **(D and J)**, BYHWD- treated **(E and K)** and BYHWD + SU5416-treated **(F and L)** groups. The immunoreactivity of p-VEGFR2 **(G)** and the number of BrdU^+^/vWF^+^ **(M)** nuclei were compared between BYHWD-treated and non-treated groups.

The effects of BYHWD on pVEGFR2 expression and angiogenesis in the brain of mice at 7 days after ICH were further examined using the double-labeling immunohistochemistry (Figure 2A-M). The results showed that pVEGFR2 expression was primarily localized to the vWF-immunoreactive ECs (Figure 2A), while pVEGFR2-positive microvessels and BrdU^+^ nuclei in vWF^+^ vessels were rarely observed in either hemisphere of the sham-operated animals (Figure 2B, H, G and M). In contrast, pVEGFR2-positive microvessels and BrdU-labeled vessels in the ICH group appeared to be dilated and most were present in the perihematomal tissue (Figure 2C, I, G and M). We observed fewer vessels positive for pVEGFR2 and BrdU in the SU5416-treated animals (Figure 2D, J, G and M). In comparison, significantly more pVEGFR2-positive microvessels and BrdU-labeled vessels could be found in the vicinity of the lesion in the BYHWD treatment groups (Figure 2E, K, G and M).

In order to verify whether BYHWD promotes VEGFR2 activation, thereby inducing angiogenesis, SU5416 was administered to ICH animals in combination with BYHWD. We found that fewer pVEGFR2-positive microvessels and BrdU^+^ nuclei in vWF^+^ dilated vessels were present around the hemotoma of the BYHWD + SU5416-treated group, compared with those of the BYHWD-treated group (Figure 2F, L, G and M).

**Figure 3:**
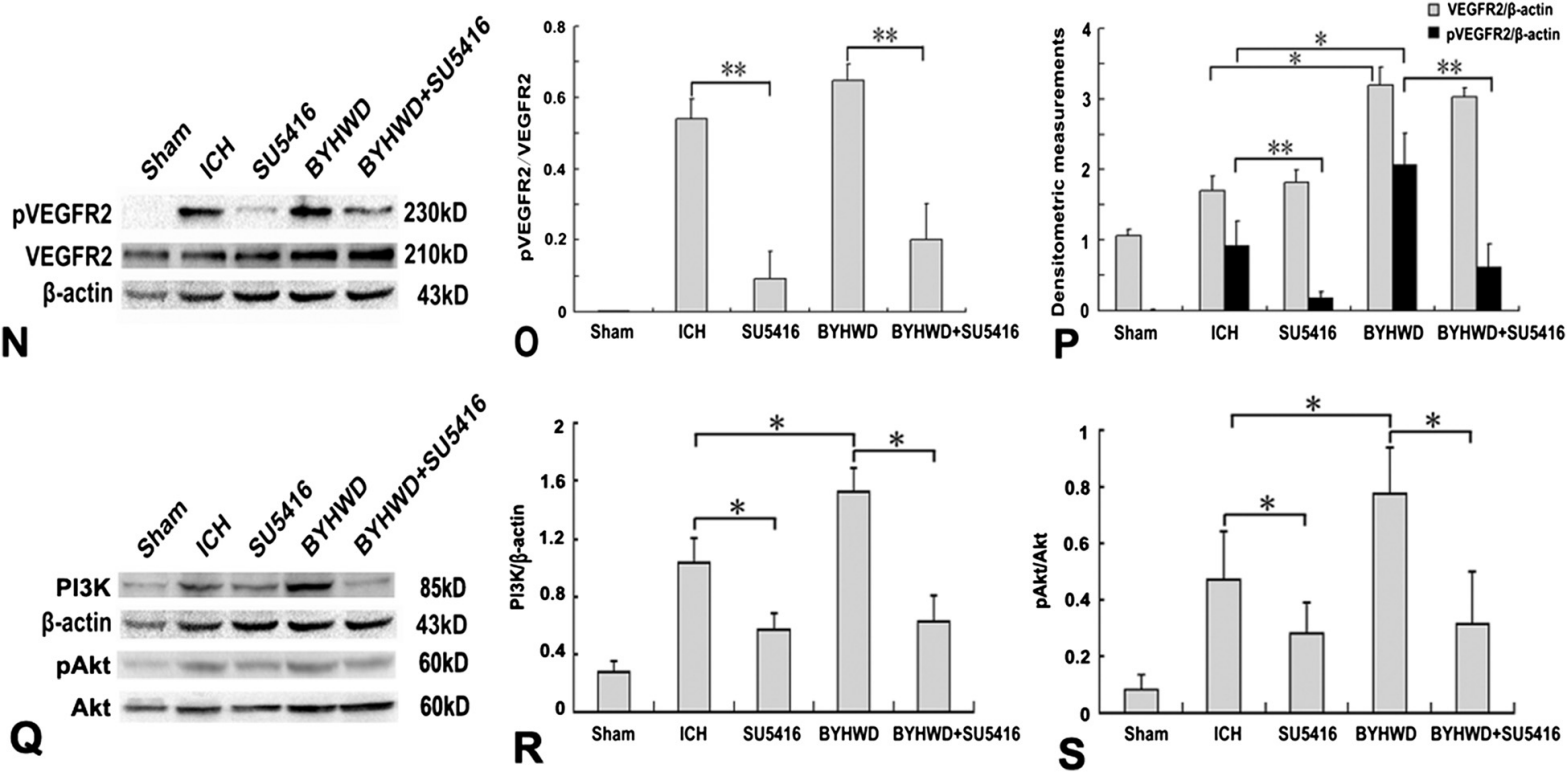
**BYHWD upregulates pVEGFR2 expression and angiogenesis via the PI3K/Akt signaling pathway.** A representative immunoblot showed that the effects of ICH, SU5416 and BYHWD on the protein levels of pVEGFR2, VEGFR2, PI3K, pAkt and Akt at the ipsilateral basal ganglion **(N and Q)**. The ratios of pVEGFR2/VEGFR2 **(O)**, pVEGFR2/β-actin, VEGFR2/β-actin **(P)**, PI3K/β-actin **(R)** and the ratios of pAkt/Akt **(S)** were compared in ICH and BYHWD with or without SU5416 (mean ± SD, n = 5, * *P* <0.05, ** *P* <0.01). Bar = 50 μm. Data were analyzed with Student’s t test and one-way analysis of variance (ANOVA), followed by Scheffe’s post hoc test.

Finally, we performed western blots to determine the effects of BYHWD on the phosphorylation of VEGFR2, and impact on the PI3K/Akt signaling pathway. The results showed that low levels of total VEGFR2 and pVEGFR2 were observed in the sham-operated brains (Figure 3N). Collagenase-induced ICH increased the activation ratio of VEGFR2, which was similar to that of the BYHWD group. This high ratio could be reversed by SU5416 (*P* <0.05, Figure 3N and O). Despite the similar activation ratio of VEGFR2 in both ICH and BYHWD administrated animals, the protein levels of total and activated VEGFR2 in the BYHWD group were about 2-fold higher than that of the ICH group (*P* <0.05, Figure 3P).

### BYHWD promoted angiogenesis via pVEGFR2 mediated PI3k/Akt signaling pathway

To further verify whether VEGFR2 activation is required for PI3K/Akt signaling transduction in BYHWD promoted angiogenesis, we examined the effect of SU5416 on the expression PI3K and the ratios of pAkt/Akt (Figure 3Q). The protein expression of PI3K and the ratios of pAkt/Akt were higher in the ICH group than that in SU5416-treated group (*P* <0.05, Figure 3R and S). These positive effects were further increased by BYHWD treatment (*P* <0.05, Figure 3R and S). However, the increased expression of PI3K and pAkt induced by BYHWD were markedly inhibited by SU5416 (*P* <0.05, Figure 3R and S).

## Discussion

Our results demonstrate for the first time that blockade of VEGFR2 phosphorylation by SU5416 could also inhibit PI3k/Akt pathway, and decrease angiogenesis in ICH mouse brains and impede the amelioration of neurological function. Importantly, our results showed that BYHWD upregulated pVEGFR2 expression and increased the activation of the PI3K/Akt pathway, thus promoting angiogenesis and accelerating the recovery of neurological function. These effects were reversed by SU5416 treatment. These data supported that the underlying mechanisms of pro-angiogenic function of BYHWD might be involved with the enhancement of VEGFR2 phosphorylation through the PI3K/Akt pathway following hemorrhagic stroke.

VEGFR-2 is a type III transmembrane kinase receptor, which is mainly expressed in vascular and lymphatic endothelial cells. The critical role of VEGFR2 is demonstrated by the fact that VEGFR2^−/−^ mice die at E8.5-9.5 due to defective development of yolk-sac blood islands, endothelial cells and hematopoietic cells [14]. Increased expression of VEGF and VEGFR2 in the basal ganglion lesion and proliferating ECs after ICH have been reported in our previous study, and their mRNAs are persistently elevated [13]. It is believed that VEGFR2 activation is a key step in regulating EC responses [29]. Suppression of VEGFR2 activity can enhance tumor regression and decrease vascular length and density as well as tumor vascularity. Additionally, inhibition of VEGFR2 signaling in cerebral ischemia and traumatic brain injury can aggravate brain injury by increasing cell death, and reducing EC proliferation [30-32]. Binding and activation of PI3K is required for phosphorylation of VEGFR2 at Tyr-801 and Tyr-1175 [33]. Thus, Akt appears to be critical for VEGF actions and a critical downstream target of PI3K. Activation of Akt was shown to promote angiogenic signals that can directly induce angiogenesis [34]. LY294002, a PI3K/Akt inhibitor, can also decrease EC proliferation and cell cycle progression [35].

To elucidate the potential role of VEGFR2 activation in angiogenesis in a mouse model of ICH, we exploited the VEGFR2 inhibitor, SU5416 in our studies. This synthetic molecule contains an unsubstituted oxindole core and a dimethyl pyrrole attached to the indolin-2-one at the C3 position. Functioning as adenine mimetics, it acts as a competitive inhibitor at the catalytic domain of the tyrosine kinase, thus blocking the interaction between VEGF and VEGFR2 [17,36]. Immunohistochemistry revealed that the pVEGFR2 immunoreactivity in microvessels and BrdU-labeled nuclei in vessels adjacent to hematoma in the ICH group exceeded those in the SU5416-administrated group. Western blot also revealed that ICH increased the total and pVEGFR2 protein levels which were further evaluated by BYHWD treatment. Although BYHWD administration had a similar activation ratio of VEGFR2 to that of the ICH, it increased almost two fold of total and activated VEGFR2 level over the ICH. Moreover, VEGFR2 activation could be reversed by SU5416 in both ICH and BYHWD treatment groups. This agreed with a prior study on experimental brain contusions showing that the specific receptor inhibitor decreases capillary density via downregulation of pVEGFR2 [31]. Protein levels of downstream signaling molecules PI3K and pAkt showed a similar trend with pVEGFR2 across all of the five groups. This data suggested that activation of VEGFR2 was necessary for PI3K/Akt signaling transduction, and consistent with the report of Fournier et al. that SU5416 administration decreased the protein level of pAkt in the rat brain with VEGF microinfusion [37]. These results altogether suggested that upregulation of pVEGFR2 and coincident activation of PI3K/Akt pathway was partly involved in the ICH-induced angiogenesis.

BYHWD, a traditional Chinese herbal prescription, has been used to treat paralysis and stroke in China for centuries [21]. We have previously demonstrated that VEGFR2 expression in brains of the BYHWD-treated group is higher than the ICH group, 7 days following stroke [24]. Radix Astragali is the main (monarch) drug in BYHWD constituting the largest concentration of 120 g. Astragaloside IV (AS-IV) is the major active constituent of Radix Astragali and has been shown to induce an angiogenic response in HUVECs by enhancing mRNA expression of VEGF and VEGFR2. This proliferation could also be suppressed by SU5416 specifically [38]. In the present study, we found more BrdU^+^ nuclei in vWF^+^ dilated vessels, and higher pVEGFR2 immunoreactivity in microvessels of BYHWD-treated animals than those in the ICH group. These results suggested that BYHWD was able to enhance angiogenesis and VEGFR2 activation. Moreover, SU5416 could reverse the effect of BYHWD on either the number of BrdU^+^/vWF^+^ vessels or the immunreactivity of p-VEGFR2. These results further suggested that BYHWD promoted angiogenic function by enhancing VEGFR2 phosphorylation following ICH.

VEGF and shear stress are two known factors that initiate the activation of VEGFR2 [39]. Our previous work showed that ICH-upregulated VEGF expression could be enhanced by BYHWD [13]. This effect has also been identified in ischemic animal models [22]. Therefore, VEGF increased by BYHWD may account for promoting VEGFR2 activation. Fluid shear stress is the other major factor that controls gene expression in vascular endothelia [40,41]. High flow (strong shear stress) drives VEGF expression to increase in parallel with its own receptor 2, and their expression presents in a time- and dose-dependent fashion [42]. Although CBF was reduced in the perihematomal zone and also remotely in the frontal cortex, this was only modest and transient. This decline, however, was resolved within 10 min and CBF restored to the normal level after ICH [43]. BYHWD was found effective in increasing capacity of microvascular reperfusion and CBF [44], which might be attributed to the increase of VEGFR2 activity.

As a sensitive and reliable indicator for detecting forelimb sensorimotor function, the mNSS tests were used to assess the neurological functions of mice, including motor ability, balancing, and alertness [45-47]. The corner turn and the food-fault test are easily performed, sensitive and objective tests for detecting impairments of sensorimotor function [45]. They have advantages on testing of multiple partial sensory and motor asymmetries associated with cortical or striatal dysfunction, including vibrissae sensory, postural and limb use biases documented in rat models [48,49]. The effects of BYHWD on neurological outcome improvement and infarction reduction in ischemic stroke have been reported [20]. In the collagenase model of hemorrhagic stroke in rats, the resulting hematoma triggers a series of events leading to secondary brain injury and severe neurological deficit [27]. As indicated by both clinical and preclinical studies, reduction of hematoma volume and decreased tissue injury improves outcomes in ICH models [50]. Other studies uncovered that angiogenic induction promotes neurogenesis, synaptogenesis, axonal sprouting and neuroblast migration to the lesion and further contributes to neurological recovery [51]. New vessel formation can improve blood supply to the injured region and regenerating neurons in the injured brain [52]. As demonstrated in our results, the improvement of neurological function in the ICH-treated and BYHWD-treated groups was coincident with the growth in the number of BrdU^+^/vWF^+^ nuclei and pVEGFR2-immunoreactive microvessels. In contrast, fewer BrdU^+^/vWF^+^ cells and pVEGFR2 labeled vessels could be observed in VEGFR inhibitor-treated counterparts, which showed a poor neurological outcome. This suggests that angiogenesis plays a central role in improving neurological function, and the pro-angiogenic effects of BYHWD may furthermore contribute to improving the phenotypes through boosting VEGFR2 activation.

## Conclusions

In summary, our results demonstrate that the activation of VEGFR2 plays a pivotal role in ICH-induced angiogenesis through the PI3K/Akt signaling pathway. Therefore, the pro-angiogenic mechanism of BYHWD in ICH, may partly involve the upregulation of pVEGFR2 through a PI3K/Akt signaling mechanism.
